# Association of Tumor Microenvironment with Biological and Chronological Age in Head and Neck Cancer

**DOI:** 10.3390/cancers15153834

**Published:** 2023-07-28

**Authors:** Martine Froukje van der Kamp, Eric Hiddingh, Julius de Vries, Boukje Annemarie Cornelia van Dijk, Ed Schuuring, Lorian Slagter-Menkema, Bert van der Vegt, Gyorgy Bela Halmos

**Affiliations:** 1 Department of Otorhinolaryngology, Head & Neck Surgery, University Medical Center Groningen, University of Groningen, 9713 GZ Groningen, The Netherlandsg.b.halmos@umcg.nl (G.B.H.); 2 Netherlands Comprehensive Cancer Organization (IKNL), Department of Research and Development, 3511 DT Utrecht, The Netherlands; 3 Department of Epidemiology, University Medical Center Groningen, University of Groningen, 9713 GZ Groningen, The Netherlands; 4 Department of Pathology & Medical Biology, University Medical Center Groningen, University of Groningen, 9713 GZ Groningen, The Netherlandsl.menkema@umcg.nl (L.S.-M.);

**Keywords:** oncology, biological age, immunohistochemistry, immunological markers, immunosenescence, tumor characteristics

## Abstract

**Simple Summary:**

There is often a mismatch between the chronological and biological age of head and neck cancer patients. Treatment is based on chronological age, while biological age seems to be a better predictor for treatment toleration. The aim of this study was to assess whether tumor characteristics are associated with chronological and biological age, and the relation with survival. We observed that, in biologically old patients, a lower infiltration of CD163+ macrophages as well as CD4+ and CD8+ lymphocytes was found in the tumor microenvironment. Chronological older patients showed significantly lower PD-L1 combined positive scores. It can be concluded from these data that biological age might have a stronger influence on tumor microenvironment than chronological age. This emphasizes the need for studies investigating the response to specific treatment regimens (e.g., immunotherapy) according to biological age.

**Abstract:**

There is often a mismatch between the chronological and biological age of head and neck squamous cell carcinoma (HNSCC) patients. Treatment is based on chronological age, while biological age seems to be a better prognosticator for treatment toleration. This study investigated whether tumor characteristics are associated with chronological and biological age. The relation with survival was also assessed. Prospectively collected data from 164 newly diagnosed HNSCC patients enrolled in the OncoLifeS database were analyzed. Biological age was assessed by a multidomain geriatric assessment. Several immunological markers were tested by immunohistochemistry on tissue microarray sections from the tumor. Disease-free survival (DFS), adjusted for chronological- and biological age, was assessed by univariable and bivariable analyses. In biologically old patients, a lower infiltration of CD163+ macrophages (*p* = 0.036) as well as CD4+ (*p* = 0.019) and CD8+ (*p* = 0.026) lymphocytes was found in the tumor microenvironment. Chronological older patients showed significantly lower PD-L1 combined positive scores (*p* = 0.030). Advanced tumor stage and perineural growth were related to a worse DFS. None of the immunological markers showed a significant association with DFS. Biological age might have a stronger influence on tumor microenvironment than chronological age. These findings should initiate clinical studies investigating the response to specific treatment regimens (e.g., immunotherapy) according to the biological age.

## 1. Introduction

Head and neck squamous cell carcinoma (HNSCC) may occur at all ages; however, the peak incidence is around the fifth and sixth decade of life [1]. Typically, these middle-aged patients are heavy smokers and drinkers or have HPV-related oropharyngeal cancer. However, patients that develop HNSCC at an older age are usually less exposed to these risk factors. It is known that age itself is a risk factor for cancer [2], and there is overlap between the genetic pathways and biochemical processes that play a role in both aging and carcinogenesis, like telomere shortening and epigenetic alterations [3]. The aging of the immune system (immunosenescence) is suggested to be an important factor in carcinogenesis in older patients [4].

Biological age, also known as frailty, describes a condition of increased susceptibility to adverse effects after a stressor event due to a progressive decline in physiologic reserves of multiple organ systems [5]. Due to their unhealthy lifestyle, HNSCC patients are often frailer, or biologically older, even compared to patients with other solid malignancies [6]. Moreover, in HNSCC patients, there is often a mismatch between chronological and biological age. Biological age is presumably better related to tumor biology than chronological age. Furthermore, biological age has been shown to be a better predictor of treatment tolerance in oncological surgery than chronological age [7].

Currently, older HNSCC patients receive comparable treatment regimens to younger patients, including radiotherapy or surgery. One of the important differences is withholding chemotherapy in patients above 70. This policy is based on a meta-analysis performed by Pignon et al., which showed no survival benefit when adding chemotherapy to treatment in older HNSCC patients [8]. However, in this meta-analysis, older patients were strongly underrepresented (357 older patients of the 17,000 analyzed cases), and non-cancer-related deaths also only included chronological age, but not biological age was determined.

A better insight into the tumor biology of HNSCC could form the basis of clinical studies on novel, age-specific treatment strategies in older patients. A recently published review proposes age-related HNSCC as a new entity besides the known (1) inherited, (2) HPV-related, and (3) traditional, substance abuse-associated pathways HNSCC [9]. It is suggested that the pathophysiology of age-related HNSCCs is based on genomic instability, cell cycle disruption, telomere shortening, and immunosenescence, leading to mutations.

Immunosenescence, defined as the gradual deterioration of the immune system associated with age, could be of importance when considering patients for immunotherapy and checkpoint inhibitor therapy. However, a recent meta-analysis showed that the chronological age-associated impairments of the immune system did not affect the efficacy of immune checkpoint inhibitor therapy [10]. Therefore, exploring biological age-related tumor characteristics could form new insights on this topic.

Thus far, only limited literature on the relation between tumor biology and chronological age has been published [10,11,12,13]. However, the relation between these characteristics and biological age has not yet been investigated. Therefore, the aim of the present study was to determine whether tumor characteristics, including immunological tumor markers, are associated with biological age. Furthermore, we aimed to determine the relation between these tumor characteristics and survival.

## 2. Materials and Methods

### 2.1. Study Design

The patients included in this study were enrolled in OncoLifeS, a prospective oncological data biobank at the University Medical Center Groningen (UMCG) (Netherlands Trial Register registration number NL7839) [14]. OncoLifeS was approved by the local Medical Ethical Committee, and this study was approved by the OncoLifeS scientific board. All patients signed informed consent before inclusion.

For this study, consecutive patients with HNSCC diagnosed between 2014 and 2016 in the UMCG were included. Patients aged 18 years and older presenting with a newly diagnosed invasive squamous cell carcinoma of the oral cavity, oropharynx, hypopharynx, or larynx were included. Patients with recurrent disease or multiple tumors in the head and neck region were excluded. Patients with HPV-related tumors (P16-positive tumors) were excluded, due to the small number of HPV-related tumors in this cohort (*n* = 20) and their distinctive biology.

### 2.2. Data Collection

Patient, tumor, and treatment characteristics were obtained from the OncoLifeS database. Patient characteristics included the age, sex, comorbidities, and outcomes of geriatric assessment. Tumor characteristics included the tumor site and stage (according to the TNM classification UICC 8th edition). Detailed histopathological information included the differentiation grade, tumor diameter, depth of invasion (doi), lymph-angioinvasion, tumor-invasive growth pattern, bone/cartilage invasion, and perineural growth.

### 2.3. Immunohistochemistry (IHC)

To construct tissue microarrays (TMAs), the representative regions of the tumor were marked on the H&E-stained slides by an experienced head and neck pathologist. Three cores of 6 µm diameter were taken from each donor block and transferred into a recipient paraffin block using the Manual Tissue Arrayer (Beecher Instruments, Silver Spring, MD, USA) [15].

**Figure 1:**
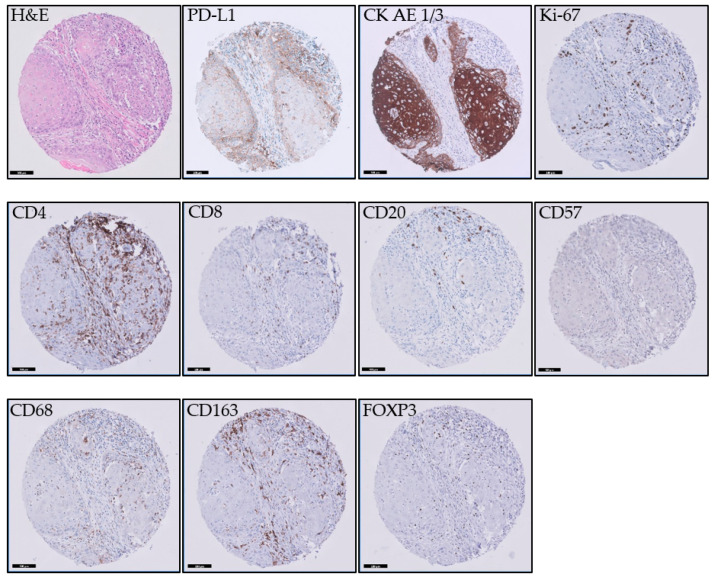
Examples of immunohistochemical staining of the different markers. The scale bar is 100 µm.

For the staining of the immunohistochemical markers, 3 µm sections were sectioned from the TMA. Sections were stained on a Ventana BenchMark Ultra immunestainer for CD4 (CONFIRM anti-CD4, clone SP35, Ventana Medical Systems, Inc., Tucson, AZ, USA), CD8 (Monoclonal Mouse Anti-Human CD8, clone C8/144B, Dako, Glostrup, Denmark), CD20 (CONFIRM anti-CD20, clone L26, Ventana Medical Systems, Inc., Tucson, AZ, USA), CD57 (Mouse Monoclonal Antibody CD57, clone NK-1, Cell Marque Corporation, Rocklin, CA, USA), CD68 (CONFIRM anti CD-68, clone KP-1, Ventana Medical Systems, Inc., Tucson, AZ, USA), CD163 (Mouse Monoclonal Antibody CD163, clone MRQ-26, Cell Marque Corporation, Rocklin, CA, USA), Ki67 (CONFIRM anti-Ki-67, clone 30-9, Ventana Medical Systems, Inc., Tucson, AZ, USA), and Pan Keratin (anti-PAN Keratin, clones AE1, AE3 and PCK26, Ventana Medical Systems, Inc., Tucson, AZ, USA). See Figure 1 for examples of immunohistochemical staining of the different markers. Visualization was performed according to the manufacturer’s protocol by using UltraView DAB. For all antibodies, antigen retrieval was performed using Cell Conditioning 1 (Ventana). The CD-8 antibody was diluted 1:20; all other antibodies were pre-diluted by the manufacturer. Sections were stained for PD-L1 (clone 22C3, Dako) on a Dako Autostainer Link 48 following the manufacturer’s protocol by using EnVision FLEX visualization system.

### 2.4. Analysis of IHC

IHC stains were analyzed using digital image analysis (DIA). The stained slides were digitized using a Philips Ultra-Fast Scanner 1.6 (Philips, Eindhoven, The Netherlands). Digital slides were stored on a central image server and loaded into the DIA platform Visiopharm Integrator System (VIS) (Visiopharm, Hørsholm, Denmark).

For each CD4, CD8, CD20, CD57, CD68, CD163, and FOXP3 IHC, an individual algorithm was developed to detect and count, respectively, the amount of T-helper cells, Cytotoxic T-cells, B-cells, natural killer cells, macrophages, infiltrating M2 macrophages, and regulatory T-cells of each core. The mean amount of positive cells was calculated from the three cores of each case. Cases were excluded from further analysis if ≥2 cores were missing or did not contain tumor cells.

Ki67 was used to determine the proliferation index. A virtual double-staining (VDS) technique was developed for this study to align the cores stained for CK-EA1/3 and Ki67. The visual verification of the alignment was performed for all cores, and alignment was manually optimized if needed. Cores were excluded from further analysis if alignment failed. After the alignment, two algorithms were used. The first algorithm was set to use the cytokeratin-stained area as the tumor classifier on the Ki67-stained core. Within the core, the complete tumor area was annotated. If present, salivary gland tissue, dysplasia, and tissue or staining artifacts were excluded.

Ki67 positivity was then analyzed with nuclear classification algorithm, which detects nuclei by morphological form and size and classifies these as positive or negative based on the pixel color and intensity. The Ki67 proliferation index was calculated by dividing the number of Ki67 positive cells by the total number of positive and negative cells within the area, classified as tumor by VDS. To compensate for intratumoral heterogeneity, the mean proliferation index was then calculated from the three cores of each case. Cases were excluded from further analysis if ≥2 cores were missing or did not contain tumor cells.

For the analysis of PD-L1, a combined positive score (CPS) of positive tumor cells and positive lymphocytes was used. A VDS technique was developed to align the cores stained for CK-EA 1/3 and PD-L1 in the same way as with Ki67 staining. The same tumor classifier was used as for the Ki67 analysis. PD-L1 positivity was analyzed with an algorithm which detects nuclei by morphological form and size and classifies these as positive or negative based on the diaminobenzidine (DAB) staining of linear structures corresponding to the membrane fragments on the tumor cells and nuclear staining in the lymphocytic infiltrate. The CPS was calculated by dividing the number of positive tumor cells and the number of positive lymphocytes by the total number of tumor cells. To compensate for intratumoral heterogeneity, the mean CPS was calculated from the three cores for each case. Cases were excluded from further analysis if ≥2 cores were missing or did not contain tumor cells.

### 2.5. Assessment of Biological Age

The biological age of the patients was assessed by a multidomain geriatric screening performed before treatment. Three domains: physical, functional, and psychological were formulated for geriatric assessment, based on the study by Bras et al. [7]. In short, the physical domain was based on the Adult Comorbidity Evaluation (ACE-27) and Malnutrition Universal Screening Tool (MUST). The functional domain was based on the Activities of Daily Living (Katz-ADL), Instrumental Activities of Daily Living (IADL) and Timed Up and Go (TUG). In OncoLifeS, mobility was added to the ADL questionnaire and financial information was not available in the IADL questionnaire. The psychological domain was based on the Mini Mental State Examination (MMSE) and Geriatric Depression Scale (GDS-15). An overview of these domains and cut-off points of the geriatric tests is shown in Table 1. Patients were considered compromised for a domain if one of the geriatric tests within the domain was impaired. When a patient was impaired in ≥2 domains (based on the accumulation of deficits theory in aging people [16]), the patient was considered biologically old. If information in one of the domains was missing or incomplete, the case was excluded from geriatric analysis.

To investigate the relation between tumor characteristics and chronological and biological age Chi-square test, Fisher’s exact test and Mann–Whitney U test were performed. To determine the relation between immunological markers and chronological and biological age, logistic regression analysis was performed by providing odds ratios (ORs), 95% confidence intervals (95%CIs), and *p*-values. The immunological markers were categorized as <median and ≥median.

For survival analysis, tumor stage was categorized in early-stage tumors (stage I and II) and advanced stage tumors (stage III and IV). Univariable and bivariable analysis were performed using the log-rank (Mantel–Cox) test and the Cox proportional hazard models. In bivariable analysis, prognostic factors were corrected for chronological and biological age. For the Cox proportional hazard models, the variables tumor diameter, depth of invasion and age were converted into categorical variables (tumor diameter cut-offs ≤20 mm, ≥21 mm and <40 mm, ≥40 mm, depth of invasion cut-offs ≤4 mm and >4 mm, age cut-offs <65 years and ≥65 years). Disease-free survival (DFS) was measured from the first consultation at the UMCG to the day of last follow-up or recurrence.

A *p*-value of <0.05 was considered statistically significant. All statistical procedures were performed with SPSS Statistics 25.0 software (IBM, Armonk, New York, NY, USA).

## 3. Results

A total of 164 patients with a median follow-up time of 36 months were eligible for inclusion. Patient-, tumor-, and treatment characteristics are presented in Table 2.

### 3.1. Tumor Characteristics

Tumor characteristics were compared between chronologically and biologically young and old patients and are shown in Table 3. In five patients, geriatric information was not complete, and they were therefore excluded from the geriatric analysis. None of the tumor characteristics were significantly associated with either chronical or biological age.

### 3.2. Immunological Markers

Table 4 shows the relation between immunological markers and the chronological and biological age. Lower PD-L1 expression was related with higher chronological age (*p* = 0.030). Expression of CD163, CD68, FOXP3, CD4, CD8, CD20, and CD57 showed no significant difference between chronologically young and older patients.

Lower CD163, CD4, and CD8 expression was related to a higher biological age (*p* = 0.036, *p* = 0.019, *p* = 0.026, respectively). The expression of CD68, FOXP3, CD20, CD57, and PD-L1 showed no significant difference between biologically young and older patients.

**Figure 2:**
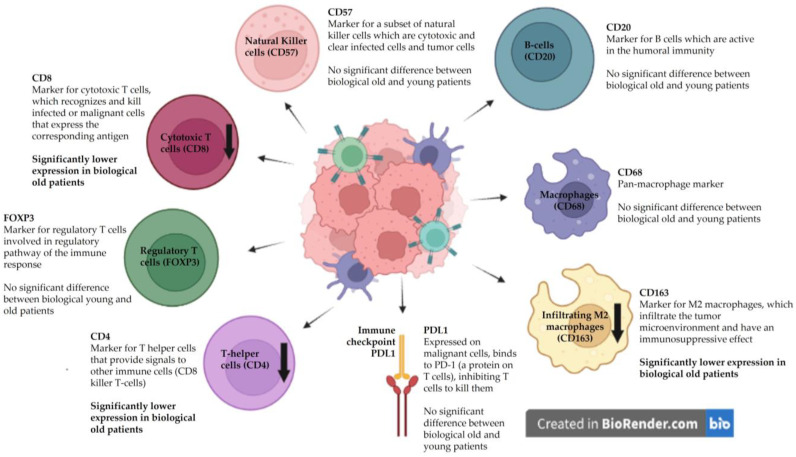
The influence of biological age on immunological markers in the tumor microenvironment. The down arrows showing lower CD4, CD8, and CD163 expression in biologically older patients.

The results on the relation between immunological markers and biological age are graphically summarized in Figure 2.

### 3.3. Survival Analysis

Uni- and bivariable results for DFS are shown in Table 5. Univariable analysis showed worse DFS in patients with advanced tumor stages (stages III and IV) compared to patients with early stage disease (stages I and II) (HR 3.293 (1.512–7.170), *p* = 0.003). Also in bivariable analysis, the advanced tumor stage showed a worse DFS, adjusted for both chronological age (HR 3.290 (1.511–7.165), *p* = 0.003) and biological age (HR 3.690 (1.614–8.439), *p* = 0.002). Perineural growth was also associated with worse DFS in the univariable (HR2.816 (1.264–6.277), *p* = 0.011) and bivariable analyses, adjusted for chronological age (HR: 2.944 (1.309–6.626), *p* = 0.009) and biological age (HR: 3.048 (1.341–6.929), *p* = 0.008). Other tumor characteristics were not significantly related to DFS in both univariable and bivariable analysis.

A similar analysis was performed for the immunological markers, shown in Table 6. None of the immunological markers showed a significant association with DFS in both univariable and bivariable analysis.

## 4. Discussion

In this study, we aimed to determine whether tumor characteristics, including immunological tumor markers, are associated with both the chronological and biological age of the patient. Furthermore, we aimed to determine the relation between these tumor characteristics and survival.

To our knowledge, this is the first study revealing a relation between biological age and tumor characteristics, including immunological markers. No age-related differences were found for histological characteristics, regarding both biological and chronological age. However, age-specific differences were found in the immunological markers, when age was assessed by biological age. In the tumor microenvironment (TME) of HNSCC in biologically old patients, lower numbers of CD163+ (type 2 or M2) macrophages, CD4+ and CD8+ lymphocytes were found. On the other hand, chronologically older patients showed significantly lower PD-L1 combined positivity scores. This implies a potential decline in tumor immune evasion in the elderly. However, it remains unclear what the exact differences are between the mechanism of chronological and biological aging. None of the immunological markers were related to survival. However, as expected, the advanced tumor stage and perineural growth were related to worse disease-free survival.

Measuring biological age can be problematic, as various measuring tools can be used to define biological age. “Comprehensive Geriatric Assessment” (CGA) is considered as the gold standard for biological age assessment. CGA is a multidimensional, time-consuming diagnostic process, performed by a geriatrician. In the clinical setting, referring all HNSCC patients to a geriatrician for performing CGA in is not feasible. Therefore, short frailty screeners have been developed to decide which patient would benefit from a CGA. However, these cannot be used to determine biological age, and their predictive value seems to be limited [17]. Determining the deficits on various geriatric domains seems to be the most reliable method for screening [7,16,18,19]; therefore, it was applied in the present study.

Immunosenescence has been described as an important factor contributing to carcinogenesis in older patients [4,20,21]. Tumor-infiltrating lymphocytes (TILs) and tumor-associated macrophages (TAMs) involved in the innate immune system are key components in the TME, playing an important role in cancer biology (Figure 2). Jeske et al. [20] also studied the TME of HNSCC patients, comparing CD4+ and CD8+ TILs and the regulatory T-cells of chronologically young and older patients measured by flow cytometry. The effect of aging on the immune system was measured in the peripheral blood lymphocytes (PBLs) of healthy volunteers and compared to the PBLs and TILs of young and older HNSCC patients. In this study, the lower frequencies and total numbers of CD8+ cytotoxic T-cells (responsible for the eradication of tumor cells) were found in older HNSCC patients. In contrast, our study found lower CD4+ and CD8+ lymphocyte infiltration only in biologically older HNSCC patients and not in chronologically older patients.

Another study investigated the effect of immunosenescence on TAMs in oral squamous cell carcinoma (OSCC) [22]. Immunohistochemistry for CD68 (a pan-macrophage marker) and CD163 (a specific M2 macrophage marker, M2 macrophages are thought to facilitate tumor growth) was comparable between patients <40 years, 40–65 years, and >65 years. All groups showed similar clinicopathological and immunohistochemical findings. The authors concluded that the similar TAM profiles in their study suggests the influence of other mechanisms, instead of immunosenescence, in young and older OSCC patients. This conclusion may be true, if only chronological age is investigated; however, we did show a significant decrease in M2 macrophages with increasing biological age, which suggests that M2 macrophages may be a contributing factor in changes in immunosenescence with increasing biological age.

Programmed Death Ligand 1 (PD-L1) is a transmembrane protein that can bind to lymphocytes. The expression of PD-L1 by tumor and/or immune cells has been linked to immune checkpoint inhibition. However, conflicting results on PD-L1 levels in HNSCC and age are reported [20,23,24]. In a recently published systematic review and meta-analysis investigating the role of PD-L1 in OSCC, no significant association between PD-L1 overexpression and age (>56, >60, >65) was found [23]. However, in two other studies performed in HNSCC patients, higher PD1 and PD-L1 expression was associated with older age [20,24]. In contrast, Ryu et al. [25] compared molecular alteration and tumor immunity in young (<45 years) and old (≥45 years) HNSCC patients and found that PD-L1 positivity was more frequent in the younger aged group (*p* = 0.01), similar to the results in this study. However, only chronological age was assessed. Based on these findings, the PD-L1 expression seems to play an important role in predicting the response to immunotherapy, irrespective of the patients’ age [23,24,26].

Stimulating the immune system opened new possibilities in cancer treatment. Since 2016, immune checkpoint inhibitors are approved for the treatment of recurrent or metastatic HNSCC [27]. The results of our study, showing lower concentrations of CD8+ T lymphocytes in biologically older patients, propose that treatment with immune checkpoint inhibitors may be less effective in this specific population. To better select patients who may benefit from the treatment with these novel therapeutic agents, the influence of biological age should be determined.

This study has some limitations. Both biopsies and surgical specimens were included in this study, resulting in low numbers of cases for specific features, like bone invasion, grow pattern, and lymph-angioinvasion when investigated on the biopsy material. Therefore, the analysis of these characteristics may not be reliable. Furthermore, the pathological tumor characteristics were analyzed retrospectively and matched with the clinical database, which may also have its impact on a relatively high number of missing data. Last, this study was performed on a limited sample size. To strengthen the results, these data should be validated on a larger scale.

## 5. Conclusions

The number of older HNSCC patients is continuously growing and there is often a mismatch between the biological and chronological age. Therefore, knowledge of biologically age-related alterations of the immune system is necessary to offer adequate treatment options for this specific group of patients. This study shows that biological age might have a stronger influence on the composition of the tumor microenvironment than chronological age. However, the exact changes in the tumor microenvironment in biological versus chronological old head and neck cancer patients needs to be further investigated. Furthermore, our findings should also initiate clinical studies, investigating the response to specific treatment regiments (e.g., immunotherapy) according to the biological age of the patients.

## Figures and Tables

**Table 1:** Geriatric screening.

Domain	Questionnaires/Assessments	Abbreviation	Cut-off Value
Physical	Adult Comorbidity Evaluation	ACE-27	None, mild, moderate, and severe
	Malnutrition Universal Screening	MUST	≥1 medium-to-high risk of malnutrition
Functional	The Katz Activities of Daily Living *	ADL	<1 considered impairment
	Instrumental Activities of Daily Living **	IADL	Female: ≤6 considered impairment Male: ≤3 considered impairment
	Timed Up and Go	TUG	≥20 impaired mobility
Psychological	Mini Mental State Examination	MMSE	≤24 considered impaired cognition
	Geriatric Depression Scale 15	GDS-15	≥6 relates to the presence of depression

* Included information on the mobility of the patients; ** Excluded financial information because not available.

**Table 2:** Baseline characteristics.

Characteristic	N (%)
Total	*n* = 164 (%)
Chronological Age	
Mean (± SD)	67.02 (10.49)
Median (p25-p75)	66.53 (41–93)
Biological Age *	
Biologically young	107 (67.9%)
Biologically old	52 (32.7%)
Sex	
Male	113 (68.9%)
Female	51 (31.1%)
Tumor site	
Oral cavity	62 (37.8%)
Larynx	61 (37.2%)
Oropharynx	31 (18.9%)
Hypopharynx	10 (6.1%)
Stage	
I	36 (22%)
II	29 (17.7%)
III	25 (15.2%)
IV	74 (45.1%)
Primary treatment **	
Surgery	83 (54.6%)
Radiotherapy	47 (30.9%)
Chemoradiotherapy	22 (14.5%)

* Missing data *n* = 5; ** missing data *n* = 12.

**Table 3:** Tumor characteristics in chronological and biological young vs. old patients with HNSCC.

		Chronological Age			Biological Age		
	Total *n* = 164 (100%)	Young < 65 y *n* = 72 (43.9%)	Old ≥ 65 y *n* = 92 (56.1%)	*p*-Value	Young *n* = 107 (67.9%)	Old *n* = 52 (32.7%)	*p*-Value
Tumor diameter (mm) median (±range)	25 (0–70) ^a^	21 (1–70) ^b^	27 (0–65) ^c^	0.138 ¶	23 (0–70) ^d^	27 (1–60) ^e^	0.462 ¶
Depth of invasion (mm) median (±range)	6 (1–55) ^f^	6 (1–30) ^g^	6 (0–55) ^h^	0.465 ¶	6 (1–30) ^i^	7 (1–55) ^j^	0.580 ¶
Differentiation grade Well differentiated Moderately Poorly	*n* = 151 30(19.9%) 115 (76.2%) 6 (4%)	*n* = 65 14 (21.5%) 47 (72.3%) 4 (6.2%)	*n* = 86 16 (18.6%) 68 (79.1%) 2 (2.3%)	0.456 ¥	*n* = 101 21 (20.8%) 75 (74.3%) 5 (5%)	*n* = 46 8 (17.4%) 37 (80.9%) 1 (2.2%)	0.735 ¥
Growth pattern Infiltrative Pushing	*n* = 81 59 (72.8%) 22 (27.2%)	*n* = 33 25 (75.8%) 8 (24.2%)	*n* = 48 34 (71.4%) 14 (28.6%)	0.800	*n* = 52 39 (75%) 13 (25%)	*n* = 25 16 (64%) 9 (36%)	0.420
Perineural growth Yes No	*n* = 128 17 (13.3%) 111 (86.7%)	*n* = 56 7 (12.5%) 49 (87.5%)	*n* = 72 10 (13.9%) 62 (86.1%)	1	*n* = 84 11 (13.1%) 72 (86.9%)	*n* = 41 6 (14.6%) 35 (85.4%)	1
Lymphangio-invasion Yes No	*n* = 130 15 (11.7%) 113 (88.3%)	*n* = 56 5 (8.9%) 51 (91.1%)	*n* = 70 10 (13.9%) 62 (86.1%)	0.423	*n* = 83 8 (9.6%) 75 (90.4%)	*n* = 42 7 (16.7%) 35 (83.3%)	0.382 ¥
Bone/cartilage invasion Yes No	*n* = 9 4 (44.4%) 5 (55.6%)	*n* = 2 0 2 (100%)	*n* = 7 4 (57.1%) 3 (42.9%)	0.444 ¥	*n* = 5 3 (60%) 2 (40%)	*n* = 4 1 (25%) 3 (75%)	0.524 ¥
Proliferation index <median ≥median	*n* = 73 41 (56.2%) 32 (43.8%)	*n* = 31 14 (45.2%) 17 (54.8%)	*n* = 42 27 (64.3%) 15 (35.7%)	0.104	*n* = 50 26 (52%) 24 (48%)	*n* = 22 15 (68.2%) 7 (31.8%)	0.201

*p*-values were estimated with the chi-square test. ¥ Fisher’s exact test performed instead of Chi-square test. ¶ *p*-values determined with the Mann-Whitney test. Tumor diameter and depth of invasion data was available only in a limited number of patient. Tumor diameter: ^a^ *n* = 104, ^b^ *n* = 43 ^c^ *n* = 61 ^d^ *n* = 68 ^e^ *n* = 34. Depth of invasion: ^f^ *n* = 61 ^g^ *n* = 24 ^h^ *n* = 37 ^i^ *n* = 41 ^j^ *n* = 20.

**Table 4:** Logistic regression analysis of the relation between immunological markers and chronological and biological age in patients with HNSCC.

		Chronological Age			Biological Age		
	Staining	*n* = 167	Odds Ratio (95%CI)	*p*-Value	*n* = 162	Odds Ratio (95%CI)	*p*-Value
Macrophages	CD163 <median ≥median	44 (53%) 39 (47%)	Ref 0.667 (0.277–1.607)	0.366	43 (52.4%) 39 (47.6%)	Ref 0.335 (0.121–0.929)	**0.036**
	CD68 <median ≥median	43 (52.4%) 39 (47.6%)	Ref 0.940 (0.388–2.274)	0.891	43 (53.1%) 38 (46.9%)	Ref 0.579 (0.218–1.537)	0.273
T-cells	FOXP3 <median ≥median	46 (54.8%) 38 (45.2%)	Ref 0.724 (0.302–1.739)	0.470	45 (54.2%) 38 (45.8%)	Ref 1.003 (0.387–2.599)	0.995
	CD4 <median ≥median	44 (53.7%) 38 (46.3%)	Ref 0.467 (0.190–1.146)	0.096	44 (54.3%) 37 (45.7%)	Ref 0.280 (0.097–0.808)	**0.019**
	CD8 <median ≥median	45 (54.9%) 37 (45.1%)	Ref 0.797 (0.328–1.934)	0.616	45 (55.6%) 36 (44.4%)	Ref 0.300 (0.104–0.866)	**0.026**
B-cells	CD20 <median ≥median	46 (22.4%) 37 (44.6%)	Ref 0.458 (0.188–1.118)	0.087	46 (56.1%) 36 (43.9%)	Ref 0.412 (0.149–1.141)	0.088
NK-cells	CD57 <median ≥median	40 (48.8%) 42 (51.2%)	Ref 0.980 (0.405–2.371)	0.965	40 (49.4%) 41 (50.6%)	Ref 0.762 (0.293–1.982)	0.577
Immune checkpoint inhibition	PD-L1 <1% ≥1%	24 (29.6%) 57 (70.4%	Ref 0.292 (0.096–0.891)	**0.030**	24 (30%) 56 (70%)	Ref 0.732 (0.260–2.059)	0.554

Biological age and chronological age were the independent factors in this analysis. *p*-values < 0.05 are marked in bold.

**Table 5:** Univariable and bivariable analyses of prognostic factors for DFS in patients with HNSCC.

	Univariable Analyses		Bivariable Analyses			
			Chronological Age		Biological Age	
	HR (95% CI)	*p*-Value	HR (95% CI)	*p*-Value	HR (95% CI)	*p*-Value
Chronological age <65 ≥65	Ref 0.871 (0.465–1.63350)	0.864				
Biological age Young Old	Ref 1.860 (0.949–3.646)	0.071				
Sex Male Female	Ref 1.333 (0.693–2.565)	0.390	Ref 1.329 (0.691–2.558)	0.394	Ref 1.416 (0.717–2.796)	0.317
Location Oral cavity Larynx Oropharynx Hypopharynx	Ref 0.685 (0.324–1.448) 1.119 (0.479–2.616) 1.230 (0.358–4.225)	0.322 0.794 0.743	Ref 0.675 (0.319–1.431) 1.100 (0.470–2.578) 1.243 (0.362–4.275)	0.306 0.826 0.730	Ref 0.594 (0.269–1.312) 1.101 (0.452–2.685) 1.386 (0.401–4.794)	0.198 0.832 0.606
Tumor stage Early stage (I–II) Advanced stage (III–IV)	Ref 3.293 (1.512–7.170)	**0.003**	Ref 3.290 (1.511–7.165)	**0.003**	Ref 3.690 (1.614–8.439)	**0.002**
Treatment Surgery Radiotherapy Chemoradiation	Ref 0.978 (0.486–1.966) 0.715 (0.247–2.066)	0.950 0.535	Ref 0.985 (0.490–1.982) 0.674 (0.228–1.997)	0.967 0.477	Ref 0.838 (0.397–1.771) 0.732 (0.251–2.131)	0.644 0.567
Tumor diameter ≤2 cm >2 cm–≤4 cm ≥4 cm	Ref 2.193 (0.862–5.577) 1.790 (0.601–5.332)	0.099 0.296	Ref 2.516 (0.959–6.600) 1.887 (0.631–5.641)	0.061 0.256	Ref 2.228 (0.874–5.681) 1.354 (0.427–4.294)	0.093 0.607
Depth of invasion ≤4 mm >4 mm	Ref 2.664 (0.866–8.193)	0.087	Ref 2.916 (0.941–9.036)	0.064	Ref 3.017 (0.971–9.375)	0.056
Differentiation grade Well differentiated Moderately differentiated Poorly differentiated	Ref 1.278 (0.561–2.912) 0.598 (0.074–4.860)	0.559 0.630	Ref 1.298 (0.569–2.960) 0.571 (0.070–4.655)	0.535 0.601	Ref 1.387 (0.574–3.352) 0.721 (0.087–5.991)	0.468 0.762
Growth pattern Pushing Infiltrative	Ref 1.981 (0.680–5.771)	0.210	Ref 2.016 (0.689–5.892)	0.200	Ref 1.934 (0.657–5.691)	0.231
Perineural growth No Yes	Ref 2.816 (1.264–6.277)	**0.011**	Ref 2.944 (1.309–6.626)	**0.009**	Ref 3.048 (1.341–6.929)	**0.008**
Lymphangio-invasion No Yes	Ref 0.474 (0.195–1.150)	0.099	Ref 0.437 (0.177–1.080)	0.073	Ref 0.462 (0.186–1.145)	0.095
Bone/cartilage invasion No Yes	Ref 0.275 (0.030–2.504)	0.252	Ref 0.348 (0.031–3.937)	0.394	Ref 0.299 (0.0.31–2.862)	0.295
Proliferation index <median ≥median	Ref 1.695 (0.702–4.094)	0.241	Ref 1.605 (0.652–3.952)	0.304	Ref 2.498 (0.988–6.313)	0.053

Results showing hazard ratios (HRs) and confidence intervals 95% (CI). In bivariable analysis, prognostic factors are corrected for chronological and biological age. *p*-values < 0.05 are marked in bold.

**Table 6:** Univariable and bivariable analyses of molecular tumor markers for DFS in patients with HNSCC.

		Univariable		Bivariable			
				Chronological Age		Biological Age	
	Immunomarker	HR (95% CI)	*p*-Value	HR (95% CI)	*p*-Value	HR (95% CI)	*p*-Value
	Chronological age <65 ≥65	Ref 0.871 (0.465–1.634)	0.864				
	Biological age Young Old	Ref 1.860 (0.949–3.646)	0.071				
Macrophages	CD163 <median ≥median	Ref 1.416 (0.612–3.277)	0.417	Ref 1.391 (0.600–3.224)	0.441	Ref 1.934 (0.797–4.693)	0.145
	CD68 <median ≥median	Ref 1.400 (0.605–3.242)	0.432	Ref 1.347 (0.580–3.130)	0.488	Ref 2.030 (0.836–4.927)	0.118
T-cells	FOXP3 <median ≥median	Ref 0.932 (0.398–2.182)	0.872	Ref 0.901 (0.384–2.113)	0.810	Ref 0.968 (0.413—2.270)	0.940
	CD4 <median ≥median	Ref 0.559 (0.234–1.333)	0.190	Ref 0.514 (0.213–1.237)	0.137	Ref 0.691 (0.284–1.683)	0.416
	CD8 <median ≥median	Ref 1.150 (0.498–2.653)	0.743	Ref 1.145 (0.496–2.643)	0.751	Ref 1.547 (0.649–3.686)	0.325
B-cells	CD20 <median ≥median	Ref 0.993 (0.429–2.298)	0.986	Ref 0.930 (0.398–2.174)	0.866	Ref 1.191 (0.509–2.786)	0.688
NK-cells	CD57 <median ≥median	Ref 0.497 (0.209–1.187)	0.115	Ref 0.499 (0.209–1.190)	0.117	Ref 0.516 (0.216–1.231)	0.136
Immune checkpoint inhibitor	PD-L1 <1% ≥1%	Ref 1.263 (0.494–3.229)	0.626	Ref 0.837 (0.419–2.929)	0.837	Ref 1.519 (0.587–3.929)	0.389

Results showing hazard ratios (HRs) and confidence intervals 95% (CI). In bivariable analysis, prognostic factors are corrected for chronological and biological age.

## Data Availability

The data can be shared up on request.
